# Bacteriophage titering by optical density means: KOTE assays

**DOI:** 10.1515/biol-2025-1209

**Published:** 2025-12-30

**Authors:** Stephen T. Abedon

**Affiliations:** Department of Microbiology, The Ohio State University, Mansfield, Ohio 44906, USA

**Keywords:** antibacterial virulence, AUC, lysis profile, titre, virulence

## Abstract

Bacteriophages, or phages, are the viruses of bacteria. Since at least the later 1940s, researchers have studied phages using spectrophotometrically determined measures of bacterial-culture turbidity (optical density). Recently, two groups have proposed the use of kinetic visualizations of phage-induced bacterial lysis to estimate phage titers (‘KOTE’ assays). Provided here is an overview of the two new publications and comparison of different approaches to interpreting resulting turbidimetric data. The latter includes especially peak culture turbidities (OD_max_) versus the timings of ‘Deviation’ of the turbidity of phage-containing curves from those of phage-free controls. Also addressed is the possible impact of the phage lysis inhibition phenotype. Overall, KOTE assays seem to provide somewhat consistent titer-estimating power, though not necessarily always with precision as high as that of plaque-based titering. This ability comes, though, at a cost of a high preliminary workload that is required to establish calibration curves. An important possibility emerging from these efforts is that OD_max_ or its timing might serve as superior indicators of phage antibacterial virulence than area under the curve measures. Additional approaches to phage titering are also reviewed, along with exploration of the long, nearly 100-year history of the KOTE technique.

## 1 Introduction

“Comparable results over a wide range of phage concentrations… suggested that… quantitative estimations of phage could be made either by determining the length of time required to produce clearing of a culture containing a certain number of bacteria at the beginning of the process or by determining the number of bacteria lysed in a given time.” Krueger [1], pp. 558–559, 1930.

“Probably more than any other single factor, it was the availability of the plaque assay that permitted the extraordinary development of bacterial virus research…” Stent [2], p. 43, 1963.

Bacteriophages, or phages for short, played key roles in the development of the field of molecular biology [3], along with making important contributions to the early study of molecular genetics. For the latter there was, for example, the Hershey and Chase [4] demonstration that DNA is the phage genetic material and Benzer’s [5] exploration of the fine structure of genes. In addition, James Watson’s graduate education was in phage biology [6] while Francis Crick provided phage-based contributions to our understanding of the genetic code [7], those researchers together serving as two of the co-discoverers of the double helical structure of DNA [8], 9]. Earlier still was the demonstration by Luria and Delbrück that mutations in bacteria, in this case to phage resistance, occur randomly rather than in response to specific external stimuli [10]. One direct result of this impact of phage research on the development of molecular biology and molecular genetics was a Nobel Prize in Physiology or Medicine awarded in 1969 to Max Delbrück, Alfred Hershey, and Salvador Luria [11]. An important contributor to this storied history was an ability to easily quantify phage numbers, i.e., their titers, particularly by employing what are known as plaque assays, e.g., [2], [12], [13], [14], [15], [16], [17], [18] and see also a number of additional methods references cited in [19]. The latter article, however, describes plaque assays as “labor intensive” – which is certainly true if large numbers of such determinations are required – while reference [20] indicates that they are “low-throughput”.

A proposed alternative phage titering approach considers kinetic phage impacts on the turbidity of broth bacterial cultures, dubbed here as Kinetic Optical density-based phage Titer Estimation (KOTE). The method has been hailed by second parties variously as “susceptible of… automation” [21], 22], “rapid” [23], 24], “inexpensive” [24], “susceptible of miniaturization” [21], 22], promising [24], “simple” [21], [22], [23], and with a potential for “high-throughput” [21], 22] (quotations are directly from [19] and/or [20]). Reviewed here are two studies independently proposing this approach, those of Rajnovic et al. [19] and Geng et al. [20], with the Rajnovic et al. stated goal to “replace routine utilization” of plaque-based phage titering “in clinical, environmental and industrial environments”. The Rajnovic et al. study has, according to Google Scholar, been cited over 100 times as of this writing; note, though, that many of those citations don’t mention their alternative phage titering approach. The more recent Geng et al. study too is gathering up citations. Thus, there exists interest in this approach.

An important limitation to this KOTE approach is that it is restricted to titering pure cultures of free virions of specific, single phage types. Indeed, it can be limited to use on specific genotypes of those single phage types, ones that have already been well characterized in terms of both their titers and the impact of those titers on the turbidity of bacterial cultures (“cannot be directly extrapolated to other bacteria/phage systems” [19]). That characterization, however, requires substantial prior investment for every phage type to be so titered. KOTE assays consequently are unlikely to be useful for applications beyond titering stocks of individual, otherwise well-studied phage types. This will tend to limit KOTE utility, for example, for environmental or industrial-contamination analyses. It is also difficult to envision use of KOTE assays for titering during one-step growth experimentation [25], [26], [27], an application for which it could be truly labor saving. This is due to KOTE calibration employing free virions rather than the phage-infected bacteria also assayed for one-step growth.

Here, KOTE assays are reviewed both historically and in terms of the viability of the technique. Overall, the contributions of the review consist of:

– Overview of techniques that have been used to titer phages (Section 2)
– Description of the basis of the KOTE technique (Section 3)
– Clarification of historical precedence for the technique (Section 4)
– Discussion of the lysis inhibition phenotype as a complicating factor (Section 5)
– Exploration of alternative metrics for scoring lysis timing (Section 6)
– Consideration of the precision of KOTE- versus plaque-based titering (Section 7)

## 2 Multiple approaches to phage titering

The word ‘titer’ comes from that of ‘titration’. For phages, this means diluting toward a point at which phages are no longer present, i.e., as equivalent to the titration of antibodies [28]. As phages are capable of replicating, however, we can distinguish phage titering approaches into total counts versus the often preferred viable counts [29]. Different approaches to total and viable counting are discussed in this section. Emphasis is on viable counting as KOTE assays too are a form of phage viable counting.

### 2.1 Phage total count determination

The oldest approach to phage total count determination is via microscopy. Electron microscopy of phages dates to 1942 [30], which Adams [31] described as a means “to determine the total number of morphologically typical phage particles in a known volume of suspension” (p. 32). Light microscopy of phage virions was described in 1945 by Hofer and Richards [32]. More recent is the viewing of virus-like particles using epifluorescent microscopy [33].

Adams [31], p. 452, described also determination of a “correlation between the size of an infectious unit as estimated by chemical methods and the particle size as determined by means of the electron microscope.” Chemical methods would estimate numbers based on protein or DNA content rather than strictly total counts. For chemical analysis, however, care should be taken to start with purified phage particles. Use of quantitative PCR avoids quantifying bacterial DNA along with phage DNA, but should still be affected by unencapsidated phage DNA unless that is first removed [34]. Anderson et al. [35] compare that and another approach to phage total-count determination to plaque counts.

What all of these techniques have in common is that if there is a desire ultimately to quantify numbers of viable phages, then there is a need for prior generation of calibration curves, as typically would be relative to plaque counts.

### 2.2 Approaches to phage viable count determination

In addition to plaque-based approaches to phage titering, e.g., [31], as well as KOTE assays as emphasized subsequently, there exist at least two other approaches to determining phage viable counts, one a variation on the other. In addition is a third approach that measures at least a component of phage viability.

#### 2.2.1 Appelmans’ method

There recently has been some emphasis on what has become known as Appelmans’ protocol. This is an approach to phage directed evolution that involves various forms of phage-phage recombination [36], [37], [38], [39], [40]. The original Appelmans’ publication [41], 42], however, is instead a description of phage titering [43].

Benefits of Appelmans’ approach are that it avoids plaquing, such as in cases where plaquing is less easily accomplished. However, it suffers from three important deficiencies. The first is that it is an inherently less precise approach to phage tittering [43]. This imprecision is in part because it is qualitative – either phages lyse cultures or they do not – and it is dependent, in its precision, on the extent of diluting employed. That is, two-fold serial diluting will provide more precision than ten-fold serial diluting. Two-fold dilutions, however, also require more effort but while still not meeting the precision obtainable with plaque-based tittering (Section 7).

The second issue has to do with phage virulence [44], [45], [46], [47], [48]. Specifically, this means that a single phage added to a bacterial culture may or may not result in culture-wide bacterial lysis, depending on starting numbers of bacteria in combination with the antibacterial performance of the phages being assayed.

The third issue is that Appelmans’ technique involves endpoint determinations. Consequently, grow-back of cultures by phage-resistant bacteria [49] can reverse the culture clearing required to assess phage presence. This equivalently is one problem associated also with efforts to determine phage minimum inhibitory concentrations (MICs) [50].

#### 2.2.2 Most probably number (MPN) method

The MPN method is a more precise variation on Appelmans’ approach. Specifically, it employs statistics – Poisson distributions – to infer phage titers from multiple tubes at a given dilution, looking for that dilution at which some but not all tubes exhibit clearing. See, however, Krueger [1] for a simpler approximation of this calculation (p. 557), i.e., “Thus if four 1 ml. portions of a 10^−6^ dilution of a phage suspension produce lysis and six do not… therefore the original lysate possesses a minimum concentration of 4 × 10^6^ phage corpuscles/ml.”

The MPN approach nonetheless suffers similarly from issues of phage virulence and endpoint determinations as that of Appelmans’ [14], 31]. So too, however, can plaquing-based approaches to phage titering suffer from issues of low efficiencies of plating [2], 31], 51], 52], which is an analogous problem. That is, these both are limitations of a phage’s ability to provide a positive signal indicating its viability, whether forming a plaque or providing the clearing of a bacterial culture. Krueger [1] also describes agreement of this approach with plaque-based titering to be “wanting”.

#### 2.2.3 Killing titers method

The killing titer approach provides a measure of phage adsorption and subsequent killing of adsorbed bacteria but not also a measure of phage viability. The assay involves adsorbing a phage population to a bacterial population, ideally to phage adsorption completion [14]. Then, as also with MPN determinations, the number of bactericidal phages present is calculated statistically. This again is based on assumptions of a Poisson distribution, though here this is in terms of surviving bacteria as plated rather than in terms of broth culture turbidity [53]. Killing titers are useful when working with phages that are still bactericidal but not necessarily either bacteriolytic or capable of producing a productive infection. For example, these may be virions carrying conditionally lethal mutations or instead which have been treated with a DNA damaging agent such as ultraviolet light [31].

Though the killing titer approach can provide reasonable precision, it does suffer from a number of shortfalls. These include of course not being able to distinguish between truly viable phages from merely bactericidal ones. In addition, the assay is less convenient to perform than plaquing due to the need for an approximation of full phage adsorption prior to plating. It also is not able to titer lower quantities of phages since phage presence is detected in terms of the fraction of bacteria that have been killed, which needs to be reasonably large to provide adequate precision. Lastly, killing titer determinations, like viable counts generally, require incubation to allow for sufficient bacterial replication to produce consistently visible, phage-free bacterial colonies.

## 3 Optical density-based phage titer estimation (KOTE)

**Figure 1:**
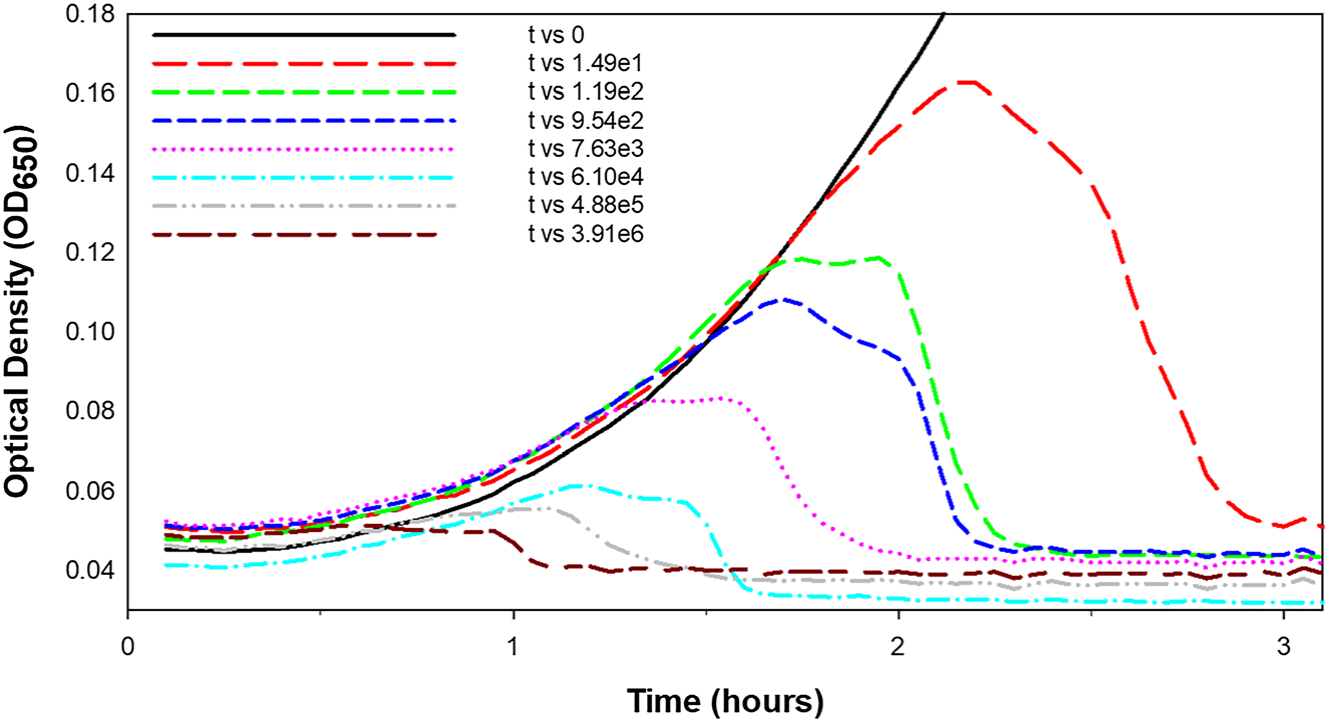
Experimental basis of KOTE assay calibration. Shown are lysis profiles made by the lysis-inhibition defective phage T4 mutant, *r48*, infecting *Escherichia coli* B. Curves are varied by starting phage titers as listed in the legend, to its right, in phages/ml units. See Supplementary Materials A, Section A3 (j_biol-2025-1209_suppl_001.docx) for methods detail and j_biol-2025-1209_suppl_002.xlsx for raw data.

A stock of a given type of lytic phage [54] – of a given titer, under a given set of broth conditions, infecting a specific initial concentration of bacteria of consistent genotype, and starting at the same point in a standard bacterial growth curve – should lyse those bacteria after a somewhat consistent interval of time; that is, should lysis indeed occur. As a result, there exists a potential for kinetic optical density-based phage titer estimation as determined from measures of the timing of this culture-wide lysis. Such KOTE assays in principle could be used as an alternative especially to most probable number (MPN)-based phage titer determinations [14]. For illustration of a KOTE-type assay, see Figure 1.

KOTE assays could be particularly useful with phages for which plaquing is challenging [55]. That includes perhaps especially phages of bacteria which are deficient in lawn formation. Alternatively, KOTE assays could be appropriate were phage research to be fully automated using robots or if substantial tittering of multiple samples of free phages is needed.

The rest of this section provides overviews of the two most recent contributions to development of KOTE-based phage titering, those of Rajnovic et al. [19] and Geng et al. [20].

### 3.1 Phage-induced culture-wide bacterial lysis (areas under curves)

Rajnovic et al. [19] established what essentially is a dose-response calibration curve [56], with the dose being the starting phage titer and the response being the impact of phages on culture turbidity over time. Rajnovic et al. quantified such responses using an area under the curve (AUC) metric, described as “the percentage of growth inhibition from integrated growth curves” and as “an attempt to make the assay quantitative”. AUCs have also been used as measures of phage antibacterial virulence [46], [47], [48] and AUC magnitude is a function of rates of phage population growth along with the timing of resulting phage-induced bacterial lysis. The Rajnovic et al. Results and Discussion section in fact places some emphasis on that timing of observed lysis, e.g., “With a decrease in OD starting at 190 min” or “Addition of 5 × 10^8^ pfu/mL resulted in a very fast decrease in optical density” (PFU meaning plaque-forming units). Rajnovic et al. thus employed AUCs at least in part as a correlate of culture-wide lysis timing.

For KOTE assays to provide reasonable titer estimations, then culture-wide bacterial lysis needs to be subsequent to multistep phage population growth. That is, it is crucial to start with sufficiently few phages, i.e., as the starting phage titer, that more than two rounds of phage infection and lysis – and thus phage population growth – precede phage infection of the vast majority of bacteria present. By contrast, if too large numbers of phages are initially provided, so that only two or even just one round of phage population growth is needed to achieve culture-wide bacterial lysis, then the timing of that lysis should be primarily a function of the phage latent period length rather than varying substantially with initial phage titer. Such an impact of too-high starting phage titers likely is the cause of the flattening of some curves in the lysis data presented by Geng et al. [20], as shown in their third Supplementary Figure. Rajnovic et al. [19] also reported a lower limit of detection of about 10^2^ phages/ml, with that number translating to “as few as 10 phage particles per assay”; keep in mind, though, that phage “detection” may be achieved at lower phage concentrations than phage “quantification”. Thus, there would appear to be both upper and lower starting-titer limits to KOTE-type assays.

Rajnovic et al. [19] otherwise employed 90 different combinations of different starting phage titers and starting bacterial concentrations toward generating calibration curves. They do not, however, appear to have tested the resulting KOTE technique using samples of previously untitered phage stocks. They do, though, indicate standard errors in the range of 1–3 % or in a few cases 4 % for individual data points. Visual inspection suggests that only relatively small sections of their individual curves – varied within curves in terms of starting phage titers – appear to be linear and curve fitting does not seem to have been attempted. These efforts thus represent an extensive pilot study, but one that is applicable only to phage T4 under specific conditions.

### 3.2 Replacing AUC with peak optical density (OD_max_)

Geng et al. [20] primarily studied phage λ, which unlike phage T4 does not display lysis inhibition (Section 5). Though phage *λ* in its wild-type form can readily display lysogenic cycles, that does not appear to have been an issue for the KOTE aspect of that study. Phages T4, T5, and P1*vir* were also studied by Geng et al. with equivalent results.

From their analysis, Geng et al. used peak culture optical density, here, OD_max_ [57], as their correlate to starting phage titers, their “Lysis OD”: “…we identified the first local maximum in the growth curve for each culture, which, for infected cultures, corresponds to the onset of massive lysis.” This metric has also been termed Maximum OD by Ghosh et al. [58], Peak by Davidi et al. [59], and Peak Density by Blazanin et al. [60].

As OD_max_ should occur just prior to the start of phage-induced culture-wide lysis, it should represent a more direct measure of the impact of phage-induced bacterial lysis on bacterial cultures than AUC determinations. This is because AUCs are not measures explicitly of lysis timing but instead are functions of a combination of (i) pre-lysis rates of phage population growth, (ii) OD_max_, (iii) OD_max_ timing, and also (iv) the rate of culture turbidity declines associated with phage-induced bacterial lysis, all of which can vary as a function of starting phage titers.

OD_max_ also is likely the most easily calculated KOTE assay-associated measurement. OD_max_ use, however, requires somewhat straightforward lysis kinetics – curves with simple shapes – which is not always the case [61], 62]; see also Supplementary Materials Section A1.1.2 (j_biol-2025-1209_suppl_001.docx) along with Figure 1. The question of whether OD_max_ is always the best correlate to the duration of phage population growth is explored further in Sections 6.

### 3.3 Taking the logarithm of starting phage titers

Geng et al. [20] found that OD_max_ varied with the logarithm of starting phage titers, as corroborating the lysis-timing findings of Krueger [1] (see also Section 6.1). Implicitly, the Rajnovic et al. [19] study also suggests this relationship (see their fifth figure). Emphasis in this section, though, is on why lower starting phage titers can diverge from this simple linear relationship, something that is suggested as well in the Rajnovic et al. data, in their case in terms of AUCs.

Linearity of relationships with the logarithm of starting phage titers should hold only so long as phage population growth is mostly linear over time on a logarithmic scale, i.e., with phages displaying exponential growth. The curves provided by Geng et al. [20], however, suggest that OD_max_ can vary from that predicted (their third Supplementary Figure). In addition to when starting with higher phage titers (i.e., Section 3.1), this is also seen when bacteria are allowed to grow to relatively high concentrations prior to substantial phage impact. Presumably this departure from linearity is due to phage growth parameters – adsorption rates, latent periods, and burst sizes [20] – changing as host bacteria begin to approach stationary phase. Geng et al. in fact explicitly state, “that when *E*. *coli* [*Escherichia coli*] growth slows down, the lytic growth rate of lambda phages decreases”.

This variation from least-squares predictions suggest a need to initiate KOTE determinations at multiple starting dilutions and/or using multiple starting bacterial concentrations, the latter an approach explicitly taken by Rajnovic et al. [19]. The goal should be to achieve acceptable accuracy for more extreme starting phage titers without having to employ complex curve fitting. This need for multiple starting dilutions [19], or for more complex curve fitting [20], should be viewed as complications on KOTE-based assays.

## 4 History of KOTE development and related concepts

The following is an historical summary of use of KOTE assays and KOTE-like experiments leading up to and then following those of Rajnovic et al. [19] and Geng et al. [20]. Supplementary Materials Section A2 (j_biol-2025-1209_suppl_001.docx) provides additional detail for those studies indicated with an asterisk:

− 1930, Krueger [1]: Likely the original KOTE assay. Used comparisons with preserved cultures of varying concentrations to score lysis timings. Found that this timing varied with the logarithm of the starting phage titer.*
− 1996, Maillard et al. [63]: Also developed a KOTE assay. Used an “automated spectrophotometric system” to document differences in the timings of peak culture turbidities as functions of different starting phage titers.*
− 2012, Turner et al. [57]: Used an automated, 96-well microtiter plate system to determine comparative phage evolutionary fitness as based especially on the timing of the end of lysis (local bacterial extinction).*
− 2013, Ghosh et al. [58]: Described “Deviation”(Section 6.2) as “1st OD change”.
− 2014, Davidi et al. [59]: Described “Deviation” as “Segregation” and noted that they were “able to identify correlation between [phage] concentration and changes in OD clearly and promptly.”
− 2015, Dalmasso et al. [64]: Explored difference in lysis timing as a function of starting phage titers, also using a 96-well plate-based assay.*
− 2018, Xie et al. [46]: Developed a 96-well phage-based phage antibacterial virulence assay involving different impacts of starting phage titers on area under the curve (AUC) measures.
− 2019, Rajnovic et al. [19]: Developed a KOTE assay employing the lysis inhibition displaying phage T4 and AUC measures, the latter as based explicitly on the Xie et al. approach.
− 2020, Storms et al. [47]: Similar to the analysis of phage virulence provided by Xie et al. but using different phage types, including phage T4.
− 2020, Konopacki et al. [48]: Similar to the analysis of phage virulence provided by Storms et al. but using different phages and refinement in AUC calculation.
− 2024, Geng et al. [20]: Similar to the work of Rajnovic et al. except using peak culture turbidities as their correlate to the logarithm of the starting phage titer.
− 2025, Su et al. [65]: Described “Deviation” as “Inflection point”.
− 2025, Blazanin et al. [60]: Further updating of optical density-based phage characterization, emphasizing the utility of “peak density, time of peak density, and extinction time”.

## 5 Lysis inhibition

The Rajnovic et al. [19] study employed coliphage T4 as its model phage, which is known to display lysis inhibition (LIN). LIN, a phenomenon which the author has been studying for 35 years [66], [67], [68], [69], [70], [71], [72], is observed in only some phages. Most notably, these are the myoviruses, T2, T4, and T6, of the original seven “Type” coliphages [62], 73]; but also the podovirus coliphage, N4 [74], and the myovirus vibriophage, ICP1 [75] (see also [76], 77]).

LIN delays lysis especially during the final round of infection during phage population growth, as it is only then that phage numbers should come to exceed bacterial numbers, resulting in a higher potential for more than phage to adsorb per bacterium [66]. Specifically, the LIN phenotype is induced by what can be described as “Secondary adsorptions” [62], though which are described by many also as secondary infections [62], 78] or instead as superinfections. The term, “Secondary adsorption”, however, may be preferable because often phages which adsorb after other phages have infected do not themselves successfully infect, i.e., as due to “Primary phage” display of superinfection exclusion [68], 79], 80].

**Figure 2:**
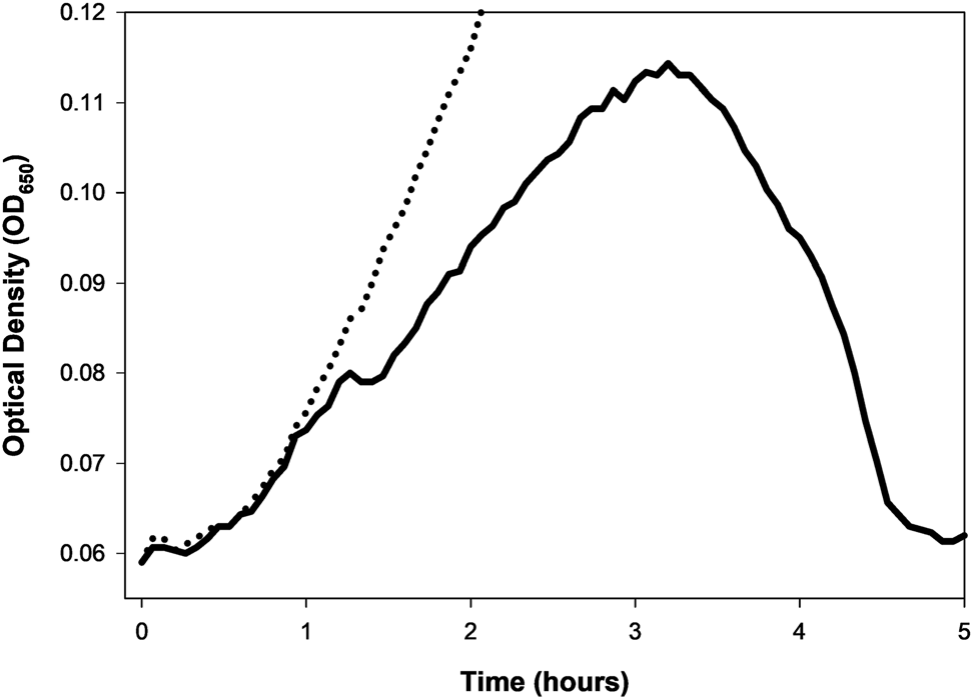
A phage T4 wild-type lysis profile. Shown are phage-free bacteria (dotted line) and phage plus bacteria (solid line), with phages added to bacteria at a low starting multiplicity at 0 h. Deviation of the phage-containing curve from that of the phage-less curve (Section 6.2) occurs around 1 h. Visually obvious lysis first occurs around 1.25 h which, without LIN, we expect would lead to complete lysis little later than after about 1.5 h (e.g., see Figure 1). Lysis of the bulk of the culture with LIN, however, doesn’t begin until around 3 h, but appears to be complete around 4.5 h. See Supplementary Materials Section A3 (j_biol-2025-1209_suppl_001.docx) for additional details on this experiment.

Initially adsorbing phages, if they are capable of displaying lysis inhibition, express LIN-required gene products [70], [81], [82], [83], [84], [85], [86]. These facilitate reception of a secondary adsorption signal, such as secondary phage DNA [87], that induces the LIN lysis delay. These delays can be substantial, ranging up to many hours relative to a typical not lysis-inhibited latent period of one-half hour or less. An example of such a delay, as following lower-multiplicity phage population growth, can be seen in Figure 2.

In Figure 2, OD_max_ occurs around 3.2 h. This compares with presumed infection of a majority of the bacteria present by around 1 h (see Section 6.2 for more on that latter point) along with a local maximum seen around 1.25 h. The subsequent decline likely corresponds to lysis of some noticeable fraction of the bacteria present, as leading to infection of any not-yet phage-infected bacteria still present and induction of LIN in the rest. Note, however, the substantial increase in OD_max_ magnitude following that local maximum, as often can occur with lysis inhibition [66], 88]. A second round of turbidity decline then occurs, following the actual OD_max_. That decline in optical density presumably corresponds to lysis of lysis-inhibited phage-infected bacteria and there takes at least 1 h to go to completion. LIN and its potential impact on KOTE determinations are considered in greater detail in Supplementary Materials A, Section A1 (j_biol-2025-1209_suppl_001.docx), as based on the experiments of Rajnovic et al. [19].

## 6 Exploring different metrics for estimating starting phage titers

Rajnovic et al. [19] employed AUC determinations while Geng et al. [20] instead used peak culture turbidities (OD_max_) toward predicting starting phage titers. Maillard et al. [63] by contrast looked at OD_max_ timing. From the latter (pages 606 and 607): “The time from the phage inoculation to the time at which the host cell growth ceased” and “Time lapse from inoculation to the initial decrease of absorbance.” So too did Krueger [1] measure lysis timing. Turner et al. [57] instead determined the timing of the end of lysis, their “extinction time”. A 2023 follow up study by the same group [89] summed up these various possibilities as “Metrics of the total bacterial population can then be extracted… including peak density, peak time, time when density drops below a threshold (‘extinction’ time), and area under the curve (AUC).” This raises the question: Which of these metrics might be preferable? In this section, especially OD_max_ and OD_max_ timings are compared for predicting starting phage titers (Section 6.1). This is followed by exploration of an additional metric, the timing of Deviation of the turbidity of phage-containing cultures from those of phage-less controls (Section 6.2 but see also Figures 1 and 2).

Because AUCs possess arbitrary start and stop timings [19], they are not similarly addressed here. That, though, does not mean that they should never be used to generated KOTE calibrations; only that their calculation possess greater degrees of freedom than Deviation, OD_max_, or the latter’s timing. Alternatively, as seen in Table 2, it is clear that lysis end (extinction) times too can provide strong correlations with starting phage titers, though further analysis of that measure also is not emphasized here.

### 6.1 Assessing correlations: OD_max_ versus OD_max_ timing, and log transformaton

Essential to KOTE analyses is for some optical density metric to correlate in some manner with starting phage titers, and ideally this correlation will be seen without complex curve fitting. From raw optical density data, OD_max_ is easily identified using the “MAX” function of Microsoft Excel^®^, and this value usually will correspond to the start of culture-wide turbidity declines of cultures (Figure 2, though see also Figure 1). One can then identify the associated timing of that peak turbidity as a measure of lysis timing. Correlations from the Rajnovic et al. [19] and Geng et al. [20] raw data (Supplementary Materials C and Supplementary Materials D and E, respectively;  j_biol-2025-1209_suppl_003.xlsx, j_biol-2025-1209_suppl_004.xlsx, and j_biol-2025-1209_suppl_005.xlsx, respectively) i.e., Pearson’s correlation coefficients (*r*) – can then be determined, including with log_10_ transformation of the various values. Results are summarized in Table 1. See Supplementary Materials F for additional details (j_biol-2025-1209_suppl_006.xlsx).

**Table 1:** Correlation coefficients (*r*) for starting phage titer versus OD_max_ or timing of OD_max_.^a^

Rajnovic et al.		Starting titer phage vs. peak turbidities				Starting phage titer vs. time of peak turbidities				Correlation	Phage titer ranges	
CFUs/ml	Phage	Lin-lin	Lin-log	Log-lin	Log-log	Lin-lin	Lin-log	Log-lin	Log-log	Max	Min	Max
5.0 × 10^7^	T4 WT	−0.91	−0.94	**−0.97**	−0.96	−0.91	−0.93	−0.94	−0.93	−0.97	5.0E + 03	5.0E + 06
2.5 × 10^7^	T4 WT	−0.93	−0.97	−0.98	−0.94	−0.85	−0.89	**−1.00**	−0.99	−1.00	5.0E + 03	5.0E + 06
1.0 × 10^7^	T4 WT	−0.75	−0.86	**−1.00**	−0.96	−0.87	−0.94	−0.96	−0.90	−1.00	5.0E + 01	5.0E + 06
5.0 × 10^6^	T4 WT	−0.80	−0.86	**−1.00**	−1.00	−0.93	−0.95	−0.96	−0.95	−1.00	5.0E + 01	5.0E + 04
1.0 × 10^6^	T4 WT	−0.76	−0.82	−0.99	**−1.00**	−0.91	−0.92	−0.98	−0.97	−1.00	5.0E + 01	5.0E + 04
1.0 × 10^5^	T4 WT	−0.84	−0.88	−0.99	**−1.00**	−0.94	−0.94	−1.00	−1.00	−1.00	5.0E + 01	5.0E + 03

**Geng et al.**		**Starting titer phage vs. peak turbidities**				**Starting phage titer vs. time of peak turbidities**				**Correlation**	**Phage titer ranges**	
**OD**_**600**_	**Phage**	**Lin-lin**	**Lin-log**	**Log-lin**	**Log-log**	**Lin-lin**	**Lin-log**	**Log-lin**	**Log-log**	**Max**	**Min**	**Max**

0.1	λ TS	−0.61	−0.78	**−1.00**	−0.96	−0.33	−0.34	−0.92	−0.94	−1.00	2.0E + 02	2.0E + 09
0.1	λ TS	−0.69	−0.82	**−0.99**	−0.96	−0.51	−0.59	−0.97	−0.99	−0.99	2.0E + 03	2.0E + 08
0.1	λ TS	−0.57	−0.89	**−1.00**	−0.88	−0.61	−0.83	−0.95	−0.90	−1.00	2.0E + 02	2.0E + 10
0.1	λ WT	−0.70	−0.84	−1.00	−0.96	−0.58	−0.66	−0.99	**−1.00**	−1.00	3.4E + 02	3.4E + 08
0.1	λ TS^b^	−0.75	−0.84	**−0.99**	−0.97	−0.55	−0.58	−0.98	−0.98	−0.99	7.4E + 03	7.4E + 08
0.1	λ TS	−0.84	−0.89	−0.99	−0.97	−0.74	−0.79	−0.99	**−0.99**	−0.99	7.4E + 03	7.4E + 07
0.1	T4 WT	−0.31	−0.55	−0.83	**−0.99**	−0.45	−0.57	−0.87	−0.89	−0.99	2.8E + 01	2.8E + 09
0.1	T5 WT	−0.46	−0.74	−0.97	−0.96	−0.48	−0.70	−0.96	**−0.99**	−0.99	2.8E + 01	2.8E + 09
0.1	P1 VIR	−0.54	−0.76	−0.97	−0.99	−0.68	−0.80	**−1.00**	−0.99	−1.00	1.6E + 01	1.6E + 09
0.1	λ TS	−0.69	−0.80	**−0.99**	−0.97	−0.51	−0.61	−0.97	−0.99	−0.99	5.5E + 03	5.5E + 08
0.1	λ TS	−0.51	−0.69	−0.97	−0.98	−0.62	−0.66	**−0.99**	−0.98	−0.99	7.4E + 02	7.4E + 08
0.1	λ TS	−0.55	−0.75	−0.98	−0.99	−0.69	−0.83	**−0.99**	−0.96	−0.99	7.4E + 02	7.4E + 08
	**Mean**	−0.68	−0.82	**−0.98**	−0.97	−0.68	−0.75	−0.97	−0.96	−0.98	n.a.	n.a.
	**STDEV.S**	0.17	0.10	0.04	**0.03**	0.19	0.17	0.03	0.04	0.03	n.a.	n.a.

^a^Greatest correlation in rows (“Max”) is indicated in bold. Phage titer ranges (starting) are those used to generate correlations, as representing more linear portions of curves. CFUs are colony-forming units. ^b^This and the following row are of the same experiment varying only in the range of starting phage titers considered. Therefore, 15 different experiments should be compared rather than 16. Considering these two curves from the same experiment separately, however, has only a minor impact on overall conclusions.

#### 6.1.1 OD_max_ utility

The most common, highest correlations appear to be found with log-transformation of starting phage titers versus OD_max_ (Table 1), without OD_max_ log transformation. This is the calculation endorsed by Geng et al. [20]. Highest correlations are seen with that calcualtion for 8 of the curves summarized in Table 1 out a total of 18. The first three curves summarized from the Geng et al. data, however, are of the same experiment, so this is actually 6 out of 16 or 37.5 %, though with a small caveat as discussed subsequently. These correlations are quite high, ranging from −0.97 to −1.00, though keep in mind that these are based on the most linear portion of these curves, as indicated in the two left-most columns of Table 1.

The next most common highest correlations can be found among the dual log_10_ transformation of the same metrics (both titer and OD_max_) with 3 of 16 or 18.8 %. These values too are quite high, ranging from −0.99 to −1.00. Often, though, the correlations seen for both of these, OD_max_ log transformed or not, have values that are within two or three hundredths, i.e., within 0.02 or 0.03.

There is one exception to the latter observation, which is the phage T4 wild-type curve of Geng et al. [20]. In that case, log-transforming OD_max_ results in a substantially greater correlation, with *r* = −0.99 versus −0.89 for the next highest. That situation changes somewhat, however, if the lowest two titer points are dropped. In that case, though *r* = −0.99 remains for the log(titer)-log(OD_max_) calculation, simply log(titer):OD_max_ improves to *r* = −0.95. Dropping just the lowest titer in turn changes these latter numbers instead to *r* = −0.98 versus −0.97. Thus, even this exception can be consistent with log(titer)-OD_max_ being a robust correlation.

#### 6.1.2 OD_max_ timing

The OD_max_ metric gives rise to the highest correlations 56.3 % of the time, 9 out of 16, which though is still somewhat less than 100 %. Alternatively, log transforming starting phage titers versus OD_max_ *timing* provides the highest correlations 43.8 % of the time, distributed between log transforming (18.8 %) and not log transforming that timing (25 %). Most of those curves are ones generated by Geng et al. [20]. As similarly seen with OD_max_, comparing with and without log transformation of these timings have values that are consistently within three hundredths (e.g., −0.99 vs. −0.96) and typically within one hundredth. In all cases, correlations based on OD_max_ rather than their OD_max_ timing are within two-hundredths with versus without phage titer log transformation.

#### 6.1.3 Take-home messages

This exercise is consistent with log transforming starting phage titers resulting in the highest correlations for calibration curves (Section 3.3), i.e., as first suggested by Krueger [1]. Though OD_max_, versus OD_max_ timing does not always yield the highest correlations, often their correlations are close. A general conclusion nonetheless is that it can be useful when generating calibration curves for KOTE assays to compare the utilities of using OD_max_ versus OD_max_ timing and also to determine whether it is more or less useful to log transform the various metrics. A more specific conclusion is that phage display of lysis inhibition, as phage T4 is the only phage tested by Geng et al. that should display this phenotype, can result in a divergent result. That divergence, however, is not seen with the T4-based results of Rajnovic et al. [19] data nor indeed when limiting the range of titers considered by Geng et al. [20].

### 6.2 Deviation as an alternative optical density-based measure

A detailed, narrative analysis of the Rajnovic et al. [19] second-figure data is presented in Supplementary Materials A, Section A1 (j_biol-2025-1209_suppl_001.docx), and summarized in Table 2. Stemming from that analysis and addressed in this section is the possibility that “Deviation” of phage-containing curves from phage-less control curves may be used as an alternative optical density-based estimator of starting phage titers. See Figure 2 for illustration of such Deviation, which happens there around the 1-h mark. See also Section 4 for “Deviation” synonyms and note that “Deviation” will often be capitalized below for emphasis.

**Table 2:** Interpretation of Rajnovic et al. [19] second-figure lysis profiles.^a^

Bacteria^b^	Phage titer^c^	Multiplicity^d^	All infected^e^	Deviation^f^	Lysis start	Lysis end	Interpretation	LIN duration^g^
10^8^/ml	5 × 10^8^/ml ◇	5	∼0 min	20 min	20 min	∼35 min	No LIN^h^	0
10^8^/ml	5 × 10^7^/ml 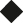	0.5	25 min	25 min	25 min	∼90 min	Weak LIN	∼65 min
10^8^/ml	5 × 10^6^/ml △	0.05	≥30, ≤60 min	*≤60 min	∼90 min	∼200 min	LIN	∼140 min
10^8^/ml	5 × 10^5^/ml ▲	0.005	70 min?	*∼70 min	∼120 min	>240 min	LIN	>170 min
10^8^/ml	5 × 10^4^/ml □	0.0005	n.k.^i^	*∼200 min				
10^7^/ml	5 × 10^8^/ml ◇	50	∼0 min	30 min				
10^7^/ml	5 × 10^7^/ml 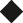	5	∼0 min	30 min				
10^7^/ml	5 × 10^6^/ml △	0.5	*60 min	*∼70 min		*∼120 min	LIN	*∼50 min
10^7^/ml	5 × 10^5^/ml ▲	0.05	*75 min	*∼90 min	*∼120 min	*∼150 min	LIN	*∼60 min
10^7^/ml	5 × 10^4^/ml □	0.005	*95 min	*∼110 min	*∼135 min	*∼180 min	LIN	*∼70 min
10^7^/ml	5 × 10^3^/ml ■	0.0005	*∼125 min?	*∼140 min	*∼160 min	*∼210 min	LIN	*∼70 min
10^7^/ml	5 × 10^2^/ml 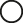	0.00005	*155 min?	*∼160 min	*∼180 min	*∼220 min?	LIN	*∼60? Min
10^7^/ml	5 × 10^1^/ml 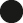	0.000005	*∼185 min	*∼190 min	*∼190 min	>240 min	LIN	>>50 min
10^6^/ml	5 × 10^8^/ml ◇	500	∼0 min					
10^6^/ml	5 × 10^7^/ml 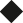	50	∼0 min					
10^6^/ml	5 × 10^6^/ml △	5	∼0 min					
10^6^/ml	5 × 10^5^/ml ▲	0.5				*150 min		
10^6^/ml	5 × 10^4^/ml □	0.05		*∼130 min	*160 min	*∼180 min		*∼50 min
10^6^/ml	5 × 10^3^/ml ■	0.005		*∼135 min	*∼175 min	*∼210 min	LIN	*∼75 min
10^6^/ml	5 × 10^2^/ml 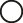	0.0005		*∼150 min	*∼190 min	*∼250 min	LIN	*∼100 min
10^6^/ml	5 × 10^1^/ml 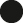	0.00005		*∼165 min	*∼210 min	*∼285 min	LIN	*∼120 min
10^8^/ml^j^	Log-lin correlation:			−0.91				
10^7^/ml	Log-lin correlation:		−0.99	−1.00	−0.99	−0.99		−0.57
10^6^/ml	Log-lin correlation:			−0.98	−1.00	−1.00		−1.00
10^8^/ml	Log-log correlation:			−0.94				
10^7^/ml	Log-log correlation:		−1.00	−1.00	−0.99	−0.98		−0.59
10^6^/ml	Log-log correlation:			0.98	−1.00	−1.00		−0.99

^a^Asterisks (*) indicate data used to calculate correlation coefficients. ^b^Starting bacterial concentration in CFUs/ml. ^c^Starting phage concentration in PFUs/ml. Symbols are from the second figure of Rajnovic et al. [19]. ^d^Multiplicity referring to MO_Iinput_, i.e., ratio of added phages to receiving bacteria. ^e^“All” refers to a guess as to when a large majority of bacteria have become phage infected. This is based in part on the starting phage input multiplicity, in part of assumptions of phage latent periods being in the range of 25 min, and in part by examination of points of deviation of phage-containing curves from phage-free curves. ^f^“Deviation” is the time point at which the phage curve deviates from the bacteria-only curve, as determined by eye. ^g^Calculated as “Lysis end” minus “Deviation” equals “LIN duration”. ^h^“LIN” = lysis inhibition. ^i^“n.k.” = No data or not interpretable (“not known”). For the sake of increased clarity, heretofore in the table, blank entries imply “n.k.” ^j^For the last six rows, the first column refers to the bacterial concentration while the second column indicates whether correlation coefficients were determined based on log_10_ transformation of asterisked data, found above in the same column or instead the straight number, indicated as “linear”, i.e., “lin”. In either case, the phage titer has been log transformed in determining the correlations coefficient (*r*), e.g., log(5 × 10^8^) = 8.7.

#### 6.2.1 Deviation plusses and minuses

**Figure 3:**
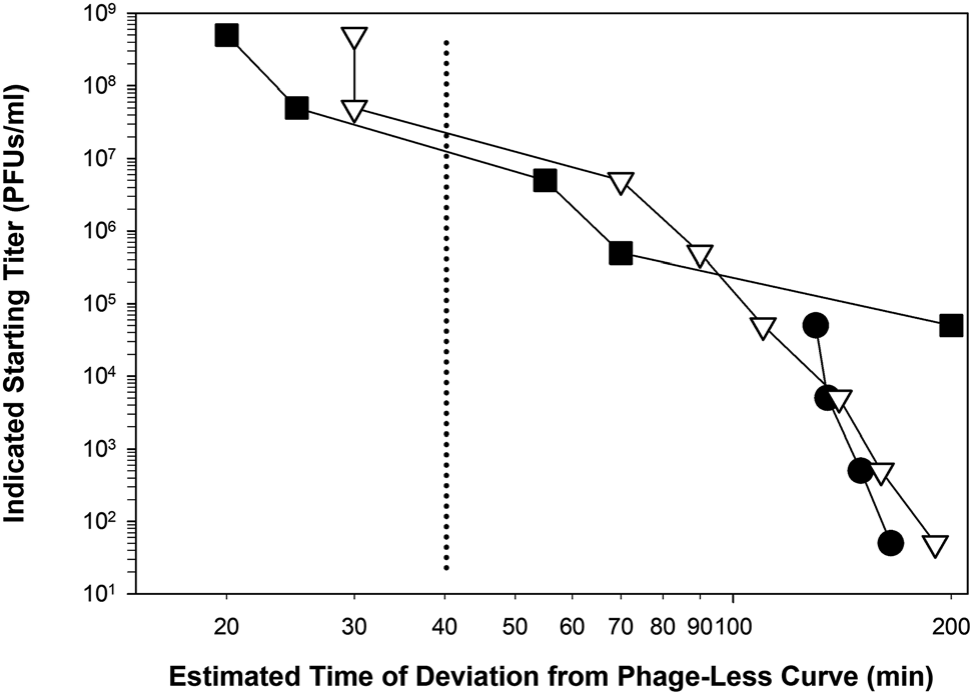
Determining phage titers from deviation of phage-containing. See Table 2’s “deviation” and “phage titer” columns for the generating data. The vertical dotted line indicates, to its left, those starting titers that were too high to allow an effective estimation of starting phage titers (PFUs/ml) (Section 3.1). Symbols differ according to starting bacterial densities: 10^8^ (■), 10^7^ (▽), and 10^6^ (●) colony-forming units (CFUs) per ml. The corresponding correlation coefficients (log-log), ignoring data found to the left of the dotted line, are *r* = −0.94, *r* = −1.00, and *r* = −0.98, respectively. Note that the “≤60 min” value found in Table 2 was set to 55 min in the figure.

A potential advantage of the point of Deviation for KOTE determinations is that it occurs earlier during assays, unless Deviation and start of lysis occur simultaneously, and certainly Deviation can occur sooner than OD_max_ for cultures that are lysis inhibited (Figure 2 and Table 2). As can be seen in Figure 3, starting phage titers also appear to vary more or less linearly with the timing of Deviation. This is particularly so when the highest starting phage titers are ignored, in this case the four data points found above 10^7^ PFUs/ml (ignored for reasons as discussed Section 3.1). Indeed, substantial correlation coefficients for these experiments can be calculated (Table 2; Figure 3).

The point of Deviation should also represent a more direct measure of durations of phage population growth, as determined by optical density means, than measures of the timing of OD_max_. This is particularly true if the latter is delayed as bacterial densities grow higher prior to substantial phage impact (Table 2 and Supplementary Materials A1; j_biol-2025-1209_suppl_001.docx) or as due to lysis inhibition (Figure 2), which can last many hours even while infecting mid-log phase bacteria [88].

Determining the point of deviation objectively nonetheless can be more challenging than determining OD_max_. A non-trivial effort therefore would likely be required to develop an effective algorithm for determining points of Deviation, especially if based on noisy data, something which is not attempted here. Still, Deviation should not be discounted completely as a potentially superior approach, at least in some cases, to describing when phage populations begin to significantly impact the presence of bacterial populations.

#### 6.2.2 Superior phage-virulence determinants?

Deviation particularly might better describe phage antibacterial virulence than AUC calculations [46], [47], [48]. This would be with that virulence defined from a perspective of the point of substantial phage adsorption of a bacterial population, which arguably is a more direct measure of the timing of phage impact on bacteria. So too, however, OD_max_ or its timing might represent superior virulence-defining metrics than AUCs. The latter especially since additional effort clearly will be needed before Deviation measures may be brought into routine use. AUC-based virulence determinations [44], [45], [46], [47], [48] meanwhile are dependent on arbitrary start and stop timings, are more readily affected by bacterial mutation to phage resistance, and, as also with the timing of Deviation, are less easily calculated than OD_max_ and OD_max_ timing.

## 7 Comparative precisions

Under the right set of starting conditions, kinetic optical density-based phage titering should be able to provide reasonable estimations of phage numbers. But even within the linear portion of calibration curves, are KOTE assays capable of attaining the precision associated simply with standard phage plaque counts? The latter theoretically are expected to have a standard deviation as low as equal to the square root of a count’s mean [31], 90]. That level of precision can be somewhat worse, however, given spotting-based plaquing, due to the low number of plaques that may be observed per spot. Carlson and Miller [91] consequently described spotting-based plaquing as only “semiquantitative”. Even so, for example, one would expect a standard deviations of just 25 % even with a mean plaque count as low as 16 (16^0.5^ = 4 and 4/16 = 0.25).

The data of Geng et al. [20], by contrast, indicate that about half of their titer-estimation tests show approximately two-fold or greater variation in titer estimation. Those authors similarly indicate that “the OD-based method can reliably distinguish unknown samples having an approximately two-fold difference in phage concentration” (also from Geng et al.: “typically within two-fold of those obtained via plating, and at worse within four-fold”; see their second figure, panels C and D). Note alternatively that Krueger [1] claimed ±5 % precision, which is consistent also with the high correlations calculated in Section 6.1, and see too the low standard errors reported by Rajnovic et al. [19] (Section 3.1).

Thus, while KOTE assays appear to be capable of high precision, there nonetheless still seems to be some potential for measures to display fairly high levels of inaccuracy.

## 8 Conclusions

The now nearly 100-year-old KOTE technique [1] requires substantial up-front efforts especially relative to those required for plaque-based titering. This is for the generation of calibration curves as well as determination of what correlations are best suited for a specific system (Section 6). For this calibration, KOTE assays clearly also require prior determination of phage titers by alternative means.

Notwithstanding those various issues, KOTE assays nonetheless may serve as a reasonable alternative to most probable number- (MPN-) based approaches to phage titering, and can do so without calibration if only relative titer information is sought [1]. Given prior calibration, KOTE assays also may be used to ballpark phage titers prior to plaquing [20], that is, to augment rather than replace plaque based titering. So too, however, may materials- or time-optimized plaquing be similarly used to ballpark phage titers, i.e., via spotting, with rapidity potentially similar to KOTE assays [92].

In summary, it seems likely that KOTE assays for many purposes will not “replace” plaque-based assays for determining free-phage viable counts. Nevertheless, KOTE-type approaches should not be overlooked as viable alternatives to plaque-based assays for making free-phage titer estimations under certain circumstances, including particularly when free-phage plaquing is technically difficult or especially laborious. KOTE utility, however, may particularly be found to the extent that fully automated on-site phage production and stock character`ization, such as for phage therapy, may be realized [93]. Lastly, the efforts presented here point to OD_max_ or its timing as possibly the most accessible metrics for defining phage antibacterial virulence. Note added in proof: for further discussion of optical density-based means of phage characterization, see Abedon [94].

## Supplementary Material

Supplementary Material

Supplementary Material

Supplementary Material

Supplementary Material

Supplementary Material

Supplementary Material

Supplementary Material

Supplementary Material

Supplementary Material
