# Supplementary material for: Bacteriophage titering by optical density means: KOTE assays
Source: Open Life Sci. 2025 Dec 30;20(1):20251209. doi: 10.1515/biol-2025-1209 (PMC13011615; doi:10.1515/biol-2025-1209)
Supplement: Supplementary file 1 — Supplementary Material [file j_biol-2025-1209_suppl_001.docx]

# Supplementary Materials A

# A1 Narrative exploration of the Rajnovic *et al.* data

The author of this review has been studying phage T4 lysis inhibition (LIN) using optical density-type experiments for 35 years [1-7]. From that experience, in this Supplementary Materials LIN as observed in the Rajnovic *et al.* [8] data is analyzed from a perspective of different phases (timings) of phage population growth, as indicated in terms of the kinetic phage impact on culture optical densities. These include (i) prior to infection of a majority of bacteria (essentially prior to Deviation; Section 6.2, main text), (ii) a pre-lysis period during which a majority of bacteria continue to be phage infected as seen both prior to and also encompassing the point of maximum culture turbidity, OD_max_, and (iii) the kinetics of subsequent phage-induced, culture-wide bacterial lysis (following OD_max_). Individual interpretations are differentiated in terms of starting bacterial concentrations (CFUs for Colony-Forming Units) and starting phage titers (PFUs, for Plaque-Forming Units). Indicated as well are starting phage “input” [9] multiplicities as derived from starting PFUs and CFUs.

The analysis is done by describing the shapes of curves as found in the second, Creative Commons Attribution Licensed figure of Rajnovic *et al.* [8]. That figure is included here in three parts to make it easier to follow the various descriptions found below. Multiple quantitative interpretations of the figure in terms of the various timings can also be found in Table 2, main text. The primary purpose of the analysis provided below is to illustrate how biological interpretation of such experiments can be relatively complex, particularly when LIN is involved, though not impossible to appreciate. Further, from this analysis came the idea of an alternative means of describing the timing of phage population growth, i.e., time of Deviation from the phage-less curve, as discussed in detail in Section 6.2, main text. It is uncertain to what extent the various interpretations provided below represent common knowledge to most phage researchers – particularly as not all phage researchers seem to be fully aware of the lysis-inhibition phenomenon – which is something that the following narrative also aims to address.

## A1.1 10^8^ CFUs/ml

### A1.1.1 Multiplicity of 5: lack of lysis inhibition

Use of lysis-inhibited phage T4 by Rajnovic *et al.* [8] presumably explains why there is a relative lack of abruptness associated with the turbidity declines of most of the curves they present (their second figure but also the various figures reprinted below). An exception to this lack of abruptness is seen when starting with 10^8^ CFUs/ml and adding 5 × 10^8^ PFUs/ml (multiplicity of 5, ◇, Figure A1.1). This combination of starting bacterial concentration and starting phage titer resulted instead in what has the appearance of a standard, not lysis-inhibited, therefore rapid lysis-like lysis profile [10-12]. That is, LIN in this specific instance does not appear to have been induced to any substantial extent. It is perhaps relevant here, and for subsequent experiments analyzed in this Supplementary Materials, that Rajnovic *et al.* appear to have initiated their experiments using stationary-phase, i.e., using just-diluted bacterial overnights, and this is rather than adding phages to log-phase bacteria. Still, despite that starting bacterial physiology, lysis at this phage multiplicity and starting bacterial concentration appears to occur following less than 30 min of incubation.


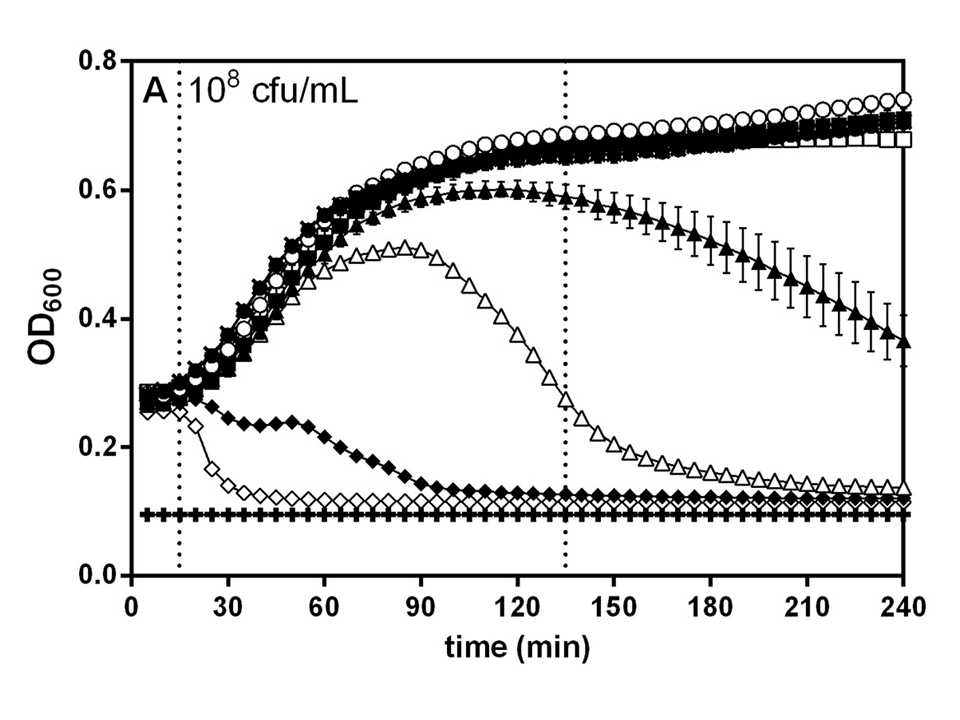


Figure A1.1. Phage T4 lysis profiles starting with 10^8^ CFUs/ ml. Symbols represent differences in starting phage titers including ◇ (5 × 10^8^ PFUs/ml), ⯁ (5 × 10^7^ PFUs/ml), △ (5 × 10^6^ PFUs/ml), ▲ (5 × 10^5^ PFUs/ml), and □ (5 × 10^4^ PFUs/ml). This figure has been copied from the 2A figure of Rajnovic *et al.* [8] which was published with a Creative Commons Attribution License “which permits unrestricted use, distribution, and reproduction in any medium, provided the original author and source are credited.”

### A1.1.2 10^8^ CFUs/ml, multiplicity of 0.5: lysis inhibition

When adding 5 × 10^7^ PFUs/ml to 10^8^ CFUs/ml of bacteria (multiplicity of 0.5, ⯁, Figure A1.1), the overall lysis profile involves an approximately 25-min initial round of infection, with 25 min representing the point of Deviation of the curve from that of the phage-less control (Table 2). This lysis, however, is not of a majority of bacteria present. Instead, less than half of the bacteria are expected to be initially phage infected, reflecting the initial multiplicity of one-half. That is, a fraction of e^-0.5^ of bacteria are expected to remain uninfected given this multiplicity, even if all added phages adsorb. This means that an estimated 60% of bacteria should have remained initially unadsorbed; see, e.g., [13-15] for further consideration of phages and Poisson distributions. This initial infection is then followed by approximately 25 min of an additional (second) round of infection. After a resulting 50 or so min, however, there is a decline in the number of phage-infected bacteria, i.e., lysis of the remaining bacteria, and this decline continues over an approximately 40-min period, as inferred by the gradually decreasing culture turbidity. An interpretation is that this curve’s shape represents a start of LIN at about 30 minutes into the experiment, with LIN then lasting for up to about 60 min, thus ending around 90 min (30 + 60 = 90 min = 50 + 40). Of additional interest, note that OD_max_ in this curve occurs more or less coincident with Deviation (around 25 min) and well prior to initiation of lysis of a majority of the bacteria present (around 50 min). This therefore is an example of a curve that does not have a simple shape (Section 2.2, main text). For additional examples of more complex curve shapes, see Figure 1, main text.

### A1.1.3 10^8^ CFUs/ml, multiplicity of 0.05: lysis inhibition

A slow turbidity decline is seen with the next-lower starting phage multiplicity (0.05, △), in this case beginning after ~90 min (also starting with 10^8^ CFUs/ml and also Figure A1.1). This culture started with only about 5% of bacteria being phage infected, i.e., since 100 × 0.05 = 5% (and e^-0.5^ = 0.95). That combination of starting phage and bacterial concentrations seems to have resulted in a slowing of the increase in culture turbidity by at most 60 min (“Deviation” in Table 2), or after essentially two ~30-min phage latent periods. That is, a small minority of bacteria were infected soon after *t* = 0 min, and then a large majority of bacteria likely became infected around *t* = 30 min, the latter as following the first phage burst. From only a fraction of the latter bacteria, LIN-inducing phages were then presumably released around 60 min. Importantly, phage T4-infected bacteria are expected to continue to increase in their optical density despite being phage infected and without ongoing bacterial division [16,17]. That, in combination with only about 5% of bacteria being initially phage infected, appears to have resulted in at best only minimal Deviation immediately after 30 or so minutes, this despite the likelihood that most of the bacteria present had presumably already become phage infected around that point in time.

This potential for culture turbidities to continue to increase despite phage infection, though this is not seen with all phage types, represents an unfortunate challenge to the general use of Deviation as a metric of phage population growth. Nonetheless, visually it seems that Deviation had indeed occurred with this curve (0.05, △, Table 2) by around 60 min, or slightly sooner, and this is even though fairly soon after 30 min it is possible that most of the bacteria were already phage infected. That is, 0.05 × 100 = 5, which is the initial multiplicity (0.05) in this case multiplied by a conservatively estimated burst size (100). Therefore, after the first round of lysis, phage multiplicity should exceed 1 even given a doubling of numbers of uninfected bacteria over that same initial 30 min, and particularly so given a burst size of greater than 100 (below).

The turbidity decline seen beginning around 90 min represents the start of a final round of lysis, though whether this is a second round or instead a third round of lysis is difficult to say. We can speculate that as a consequence of a presumed variability in the timing of lysis displayed by individual lysis-inhibited, phage-infected bacteria – i.e., some phage-infected bacteria lyse sooner than others – that it then takes a total of about 60 to 90 min until this lysis is completed, starting as indicated around *t* = 90 min. LIN thus seems to last for some of these phage-infected bacteria for well over 100 min, if starting around *t* = 60 min and ending as late as around *t* = 200 min into the experiment, or perhaps starting even as early as around 30-min after the addition of phages as phage multiplicities could have exceeded one even at that point.

### A1.1.4 10^8^ CFUs/ml, multiplicities of 0.005 and 0.0005: lysis inhibition

With 5 × 10^5^ PFUs/ml as the starting phage titer, again with a starting bacterial concentration of 10^8^ CFUs/ml (multiplicity = 0.005, ▲, Figure A1.1), lysis as a turbidity decline seems to begin roughly around 120 min while complete lysis seems to occur *more than* 120 min later (that is, greater than 240 min minus 120 min is greater than 120 min). Complete lysis, in other words, presumably would have occurred well after experiments had been terminated at 240 min, with the overall delay in lysis (starting around 120 min and continuing at least through 240 min) likely due to LIN in combination with a gradual lysis of lysis-inhibited bacteria.

At least some of these post-120-min lysing bacteria may have become infected as early as 60-min. Specifically, with a burst size of 100, then after one round of infection, lysis, and subsequent adsorption, somewhat less than half of the bacteria present would be expected to become phage infected, i.e., 100 × 0.005 = 0.5, as occurring around 30 min. Keep in mind, though, that uninfected bacteria are expected to have continued to replicate during that first phage latent period, resulting in an expectation of less than half of the bacteria becoming phage infected at this point given that burst size, but which could be a larger fraction if the burst size instead were higher as indeed one could expect for phage T4 [18]. The rest of the bacteria present would then become infected after some of those subsequently infected bacteria have lysed (second round of lysis), perhaps prior to 70 min (e.g., around 60 min), which is where Deviation seems to occur. A majority of those now-phage-infected bacteria then do not lyse until somewhat later, a delay presumably due to LIN, i.e., with the final round of lysis then starting around 120 min.

In comparing the 0.005, 0.05, and 0.5 multiplicity curves at this same starting bacterial concentration (10^8^ CFUs/ml), it seems that with increasingly lower starting phage multiplicities – and thus lower starting phage titers – progressively longer delays are seen until lysis is more or less complete. These longer delays until lysis is complete in turn seem to correspond to the increasingly higher culture turbidities seen at the point where phage-infected curve turbidities deviate (i.e., Deviation) from those of phage-uninfected curves (corresponding to around 70 min for the 0.005 multiplicity curve). Thus, for starting phage multiplicities of 0.5, 0.05, and 0.005, roughly 65 min, 140 min, and greater than 170 min periods of LIN may be seen, respectively.

Starting with 5 × 10^4^ phages/ml (multiplicity = 0.0005, □, Figure A1.1), noticeable Deviation does not seem to occur until about 200 min following the point of phage addition. This is just 40-min short of termination of the presented experiment. It therefore is impossible to tell from the presented curve what the timing of lysis might have been nor whether LIN would have occurred.

## A1.2 10^7^ CFUs/ml

### A1.2.1 Multiplicities of greater than 1: no meaningful data

Starting instead with 10^7^ CFUs/ml, the starting phage titers of both 5 × 10^7^ (⯁, Figure A1.2) and 5 × 10^8^ (◇, Figure 2A) PFUs/ml represent multiplicities of greater than 1 (5 and 50, respectively). This had the effect of preventing both measurable rises and measurable drops in culture turbidities. The former, lack of rise in turbidity, should be due to the high phage multiplicities added, presumably as equivalent to the 10^8^ CFUs/ml and 5 × 10^8^ PFUs/ml multiplicity of 5 curve (◇) seen in Figure A1.1. The latter, lack of a measurable drop in turbidity, however, instead would be due also to the relatively low starting bacterial concentration. These effects, in this case, seem to be independent of whether or not LIN had occurred.


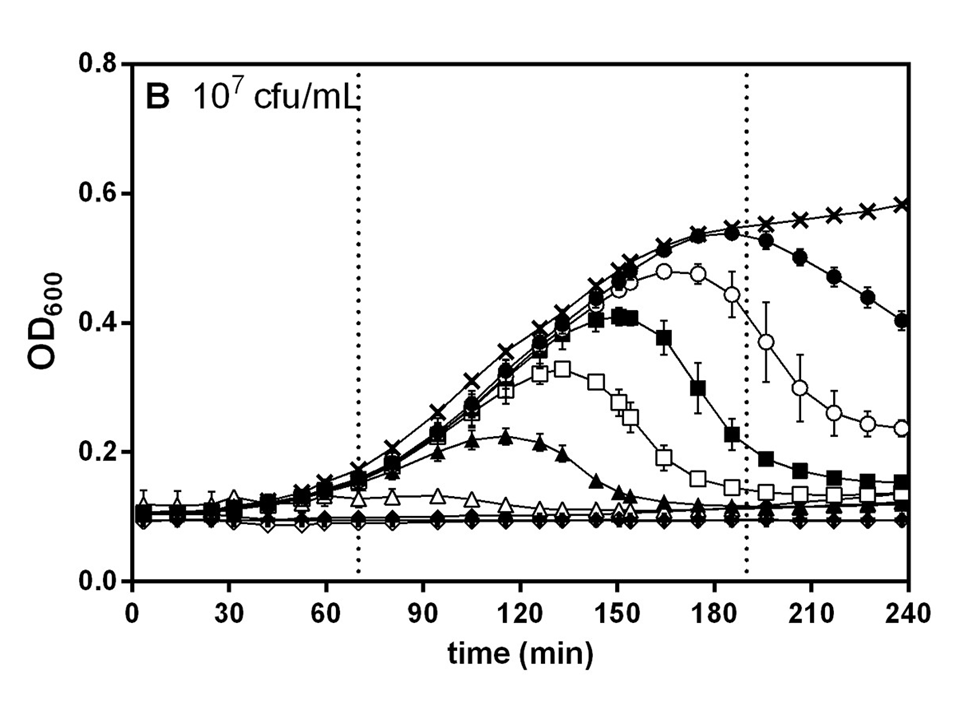


Figure A1.2. Phage T4 lysis profiles starting with 10^7^ CFUs/ ml. Symbols represent differences in starting phage titers including △ (5 × 10^6^ PFUs/ml), ▲ (5 × 10^5^ PFUs/ml), □ (5 × 10^4^ PFUs/ml) , ■ (5 × 10^3^ PFUs/ml), ○ (5 × 10^2^ PFUs/ml), and ● (5 × 10^1^ PFUs/ml). This figure has been copied from 2B figure of Rajnovic *et al.* [8].

### A1.2.2 10^7^ CFUs/ml, multiplicities of less than 1: lysis inhibition

With 5 × 10^6^ phages/ml and starting with 10^7^ CFUs/ml (multiplicity 0.5, △, Figure A1.2), the culture appears to have gone through two rounds of infection prior to Deviation as evidenced by Deviation’s 60-min and therefore approximately two-fold latent-period timing. This was then followed by seemingly a bit of lysis, seen as a small dip in culture turbidity coincident to the left-most dotted vertical line shown in the figure (A1.2, around 70 min). This dip in turbidity is then followed by about 50 min of what appears to be ongoing phage infection prior to completion of culture-wide lysis, as is mostly concluded by around 120 min. That delay in lysis presumably represents a lysis-inhibited latent period that lasts about 50 min for at least some of the phage infections present. Note, though, that this specific curve is more easily appreciated if emphasis is placed on it (the phage-containing curve) rather than on the turbidity of the phage-uninfected bacterial culture, as exemplified in Figure A1.3 vs. Figure A1.2.


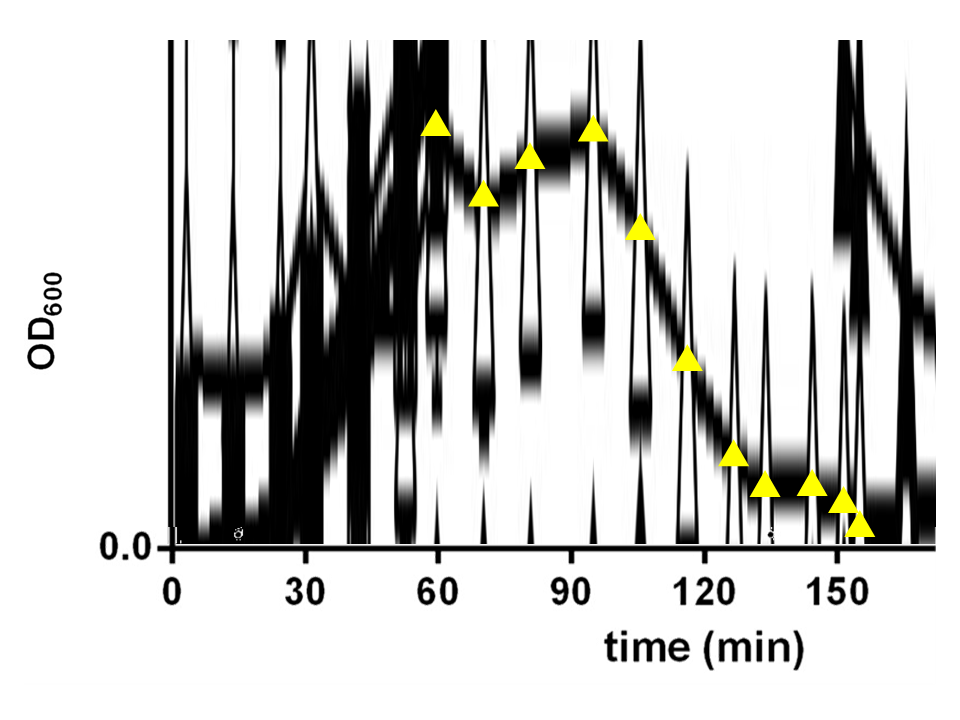


Figure A1.3. Close up of phage T4 lysis profiles starting with 10^7^ bacteria (CFUs) per ml, multiplicity 0.5. Stretched symbols are those of △, referring to 10^6^ PFUs/ml, which have been overlain with yellow, closed triangles to improve visualization of approximate centers of the stretched symbols. The curve in question is most easily observed starting a little after 60 min, which is indicated with the first shown yellow triangle going from left to right. As can be seen, the apparent beginnings of a dip in optical density is observed there, which is then reversed (slope becomes positive) around 70 min into the experiment, and which is followed by a peak in optical density (OD_max_) between 90 and 100 minutes. From this manipulation, one can also see that the decline in culture turbidity perhaps continues until at least 150 min into the experiment. The shape of this curve, as presumably representing phage T4 LIN, seems to correspond to that of a typical phage T4 lysis profile, e.g., [1,2,5,6] as well as Figure 2, main text, and Figure A1, above. More difficult to see in this rendering, however, is what the curve looks like prior to deviation, which at this scale is well above the zero optical density on the figure’s left. This latter, inexplicable aspect of the curve is nearly unnoticeable in the original figure (seen here as Figure A1.2). The figure itself was copied from the 2B figure of Rajnovic *et al.* [8] and then manipulated into its present form using Microsoft PowerPoint®.

Though less easily interpreted, with 5 × 10^5^ phages/ml (multiplicity 0.05, ▲, Figure A1.2), the cultures appear to go through between two and three rounds of infection and lysis before Deviation, though this may be just two rounds of infection assuming delays associated with slower virion adsorption, i.e., given the ten-fold lower starting bacterial concentrations vs. 10^8^ CFUs/ml (Figure A1.2 vs. Figure A1.1). The apparent start of lysis (~120 min) again appears to be followed by roughly 60 min until completion. In fact, for this set of experiments, all starting with 10^7^ CFUs/ml, with each ten-fold reduction in starting phage titers there appears to be a roughly 20-30 min greater delay until the phage-containing culture Deviates from that of the phage-free culture, which is then followed by 60 or so minutes of LIN.

Peak turbidities starting with 10^7^ CFUs/ml generally seem to be somewhat lower than what is seen with a starting bacterial concentration of instead 10^8^ CFUs/ml, probably explaining the somewhat shorter durations of LIN at this lower staring bacterial density. Those shorter durations of phage infections in turn allow for estimation of their lengths including that of LIN. This inferred length of LIN seems to remain somewhat similar between 50 and 70 min for multiplicities of 0.05 (▲), 0.005 (□), 0.0005 (■), and 0.00005 (○). Further, this LIN-duration data (Table 2) suggests that with each ten-fold decrease in starting phage titer, the extent of LIN increases by 10 minutes, from ~50 (▲) to ~60 (□) and then to ~70 (■) min. The ~70-min duration (multiplicity of 0.00005; ○), however, is potentially longer given that the experiment was truncated at 240 min. There thus seems to be an increasing length of LIN associated with later Deviations and thereby higher overall culture turbidities, just not as dramatically longer with each step when starting with 10^7^ CFUs/ml in comparison to that seen when starting with 10^8^ CFUs/ml.

With the resulting increased potential to compare those durations, we see what would appear to be lengths of LIN that are fairly predictive of starting phage titers of 5 × 10^5^, 5 × 10^4^, and 5 × 10^3^ PFUs/ml, respectively. Specifically, in log transforming starting titers, these pair up as 5.7 log_10_ PFUs/ml and 50 min, 4.7 log_10_ PFUs/ml and 60 min, and 3.7 log_10_ PFUs/ml and 70 min, with *r* = -1.00 (*r* = sample correlation coefficient, also known as Pearson's correlation coefficient, and which is calculated using the “CORREL” or equivalent function of Microsoft Excel®). This perfect correlation, though, comes with the caveat that lysis-inhibition duration was not determined necessarily with high accuracy. Workflow nonetheless consisted of duration estimation followed by *r* calculation rather than the converse. That is, there was no *post hoc* manipulation involved in the above effort.

## A1.3 10^6^ CFUs/ml: lysis inhibition

Similar interpretations can be made starting with 10^6^ CFUs/ml (Figure A1.4) rather than with 10^7^ CFUs/ml (Figure A1.2), except that input multiplicities are ten-fold higher for a given starting phage titer and peak turbidities are generally lower. Together, these have the effect of making the assay more suitable for lower starting phage titers, as due to lower starting bacterial concentrations. They also have the effect of making the assay less suitable for higher starting phage titers as due to somewhat small resulting changes to culture turbidities. The latter, re: Shymialevich *et al.* [19], are as associated with phage-induced bacterial lysis. That is, phage-induced drops in culture turbidities are nearly non-existent, or at least not discernable, given higher starting phage multiplicities. The opposite is true for starting bacterial concentrations of 10^8^ CFUs/ml, which were less appropriate for lower starting multiplicities but more suitable for higher starting concentrations (see Table 2, main text).


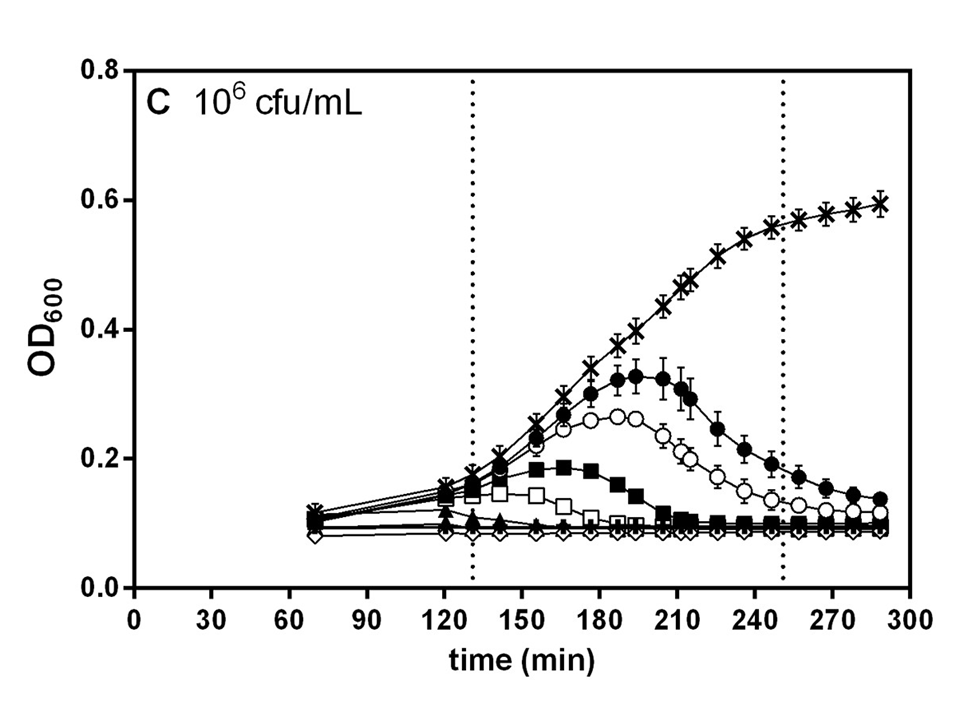


Figure A1.4. Phage T4 lysis profiles starting with 10^6^ CFUs/ ml. Symbols represent differences in starting phage titers including ▲ (10^5^ PFUs/ml), □ (10^4^ PFUs/ml) , ■ (5 × 10^3^ PFUs/ml), ○ (5 × 10^2^ PFUs/ml), and ● (5 × 10^1^ PFUs/ml). This figure has been copied from 2C figure of Rajnovic et al. [8].

The existence of LIN clearly leads to quite long durations of lysis rather than what should be more desirable abrupt declines in culture turbidities, i.e., for the sake of estimating starting phage titers (KOTE assays, see the main text). On the other hand, and unlike starting with 10^7^ CFUs/ml, the estimated increase in the length of LIN with decreasing starting phage multiplicities seems to be quite clear, going from 50 to 75 to 100 to 120 min (Table 2), with an ability to gather a more complete data set at this starting bacterial concentration in part attributable to these experiments having been run for a total of close to 300 min rather than only 240 min.

## A1.4 Lysis inhibition and the rapidity of assays

A 300-min assay duration, in combination with necessary initial preparation and subsequent data analysis, would likely not be compatible with obtaining starting titer estimations fast enough to allow same-day use of that information. This is relative to being able to obtain plaque-assay results at the start of the next day as following, in most cases, just an overnight incubation. Specifically, the time advantage of KOTE assays over plaque assays would require a doubling up of efforts over the course of a single day so that titer-estimation results may be used without waiting until the next day. Alternatively, this issue might be overcome by shortening assay incubations to, for example, three hours instead of four or five, and using something other than areas under curves toward estimating starting phage titers (main text). This shortening of incubations, however, appears to be less easily achieved if waiting for lysis given phage display of LIN with its extended delay until peak culture turbidity, and particularly so since lysis inhibition by other phages can be even longer [7,20], hence a potential utility to use of Deviation instead of OD_max_ or its timing as a correlate to starting phage titers (Section 3, main text).

# A2 Historical precedence for KOTE development (details)

KOTE-like techniques appear to have been described by various researchers prior to the 2019 and 2024 publications by Rajnovic *et al.* [8] and Geng *et al.* [21]. That historical ‘foreshadowing’ is considered in this section. See Section 4, main text, for a more complete exploration of historically relevant KOTE-like publications.

## A2.1 Krueger (1930): there is nothing new under the sun

Though not an automated optical density-based approach, Krueger [22] describes essentially the same method as KOTE assays, but with readings made by comparing culture turbidities to those of “formalinized bacterial suspensions of known cell concentrations” (p. 560). He further describes the accuracy of his approach as “well within ±5 per cent” (p. 563).

Adams [23], in 1959, provided a fairly comprehensive summary of the Krueger [22] study as follows (p. 31):

A kinetic method of phage assay was developed by Krueger [22], for use with rapidly lysing phages, which gives precise and reproducible results within certain limits. The procedure is to add appropriate dilutions of phage to tubes containing standard suspensions of actively growing host cells, and to determine the length of time required for lysis to reduce the bacterial turbidity to an arbitrary end-point. The time of lysis is inversely proportional to the logarithm of the initial phage concentration over a considerable range. A standard phage preparation is assayed in parallel with the unknown and the activity of the unknown is expressed in terms of an arbitrary unitage defined for the standard. One defect of the method is that it does not give the assay in terms of infectious phage particles but only in amounts relative to the standard.

## A2.2 Maillard *et al.*, 1996: an actual KOTE assay (OD_max_ timing)

Maillard *et al.* [24] represents an additional earlier observation of the impact of starting phage titers on phage lysis kinetics. Their interest was on the “Virucidal activity… of biocides” [25]. These authors used an optical density-based assay that both was automated – though didn’t employ microtiter plates – and was used as a means of assessing biocide-mediated decreases in phage titers. This they described as an “*indirect* assessment of phage survival” (emphasis added; p. 605). Of interest, they (p. 606) noted that

The plaque assay… is time-consuming and requires relatively long incubation times (usually between 18 and 48 h). Potentially this could be improved by the use of an automated spectrophotometric system, which monitors the characteristic phage-induced bacterial host lysis curve, in broth culture.

Their second figure explicitly shows, at least to a degree, an increasingly late timing of phage-induced culture-wide bacterial lysis with declining start phage titers. It is of interest, though, that three of the middle starting phage titers, 10^6^, 10^5^, and 10^4^ PFUs/ml, all displayed a very similar OD_max_ as well as similar timings of those peaks – i.e., OD_max_ timings – suggesting limitations to the KOTE technique in at least this system (for the latter, these are approximately 44, 47, and 49 min vs. 20 and 75 min for the next lower and higher starting phage titers, respectively). These three middle curves nevertheless displayed consistently increasing AUCs with decreasing starting phage titers, as estimated by eye, and this was due to a slowing of lysis-associated turbidity declines given lower starting phage titers. Perhaps, therefore, the most useful metrics used as surrogates for starting phage titers may differ between systems (see also Section 3).

The authors also noted that, “The phage particles pretreated with these biocides presented a degree of inactivation which was different from the results obtained with conventional plaque count assay” (p. 608) and (p. 609), “The basis of these differences must be clearly established before this method may be applied to routine viricidal evaluation.” Maillard *et al.* [24], notwithstanding those concerns, should be viewed as an earlier and perhaps even the earliest development of a phage-based KOTE-like assay.

## A2.3 Turner *et al.*, 2012: (end of lysis)

Turner *et al.* [26] employed optical density as a means of inferring phage properties. Their approach was to start with a known phage titer (4 × 10^2^ PFUs/ml) and then to use optical density-based lysing-timing information to compare phage properties. Considered particularly was that of phage evolutionary fitness. For the latter, see [21,27] for newer, somewhat equivalent emphases. Though this was rather than to infer starting phage titers, their model nonetheless should be viewed as an alternative approach to associating initial phage titers with resulting phage-induced turbidity reductions of bacterial cultures.

The Turner *et al.* approach is of further interest because of their determination of the endpoint of phage-induced culture-turbidity declines: “To measure *t*_ext_ [‘time at which bacteria become extinct’] more accurately, we used the first time point which was more than 80 min after the peak OD_600_, and which differed by less than 0.002 from the preceding measurement.” This bacterial ‘extinction’ occurred around ODs of about 0.12, so this could be a change from, e.g., 0.122 to just 0.121 between time points.

A 2023 follow-up, theoretical work from this group placed some emphasis instead on OD_max_ {Blazanin, 2023 45014 /id}. Of interest, there they also noted that “growth curve metrics can be used to quantify general phage infectivity, but are unlikely to be useful to quantify specific phage life history traits like adsorption (cell-attachment) rate, burst size, or lysis time.” See equivalently, {Blazanin, 2025 45389 /id}.

## A2.4 Dalmasso *et al.*, 2015, variation with starting phage titer (microtiter plates)

Dalmasso *et al.* [30] presented an experiment that is similar to that of Maillard *et al.* [24] in terms of exploring the impact of different phage titers on the turbidity of a bacterial culture over time (see also the curves presented later, 2018 and 2020, by Xie *et al.* [31] and Storms *et al.* [32]). Dalmasso *et al.* however was a microtiter plate-based study, as too was the earlier Turner *et al.* study [26], both contrasting the approach used by Maillard *et al.* [24]. For the latter, p. 605, instead, “A conical flask containing 150 ml of [nutrient broth] was connected to a spectrophotometer cuvette, linked to an autofill system… Every minute the culture absorbance was recorded spectrophotometrically at 500 nm.”

# A3 Lysis inhibition

## A3.1 Complete legend for Figure 2, main text

See next page, top, for duplication of Figure 2 as found in the main text.

Figure 2. A phage T4 lysis profile, the same phage as used by Rajnovic *et al.* [8]. The curves – dotted line for bacteria-only and solid for with phage – each represent only a single technical repeat (one of three performed in parallel). This was done to better align their lower portions, an agreement of time points which is not always as obvious between technical repeats. Deviation (Section 6.2) of the phage-containing curve from the phage-less curve occurs here at or after 60 min, which we can hypothesize should correspond to the occurrence of phage infection of a majority of the bacteria present. Around 1.25 hours (75 min), the first turbidimetrically visible round of lysis appears to have occurred. This does not lead to substantial overall lysis of the culture, however, as maximum culture turbidity (OD_max_) is seen nearly two hours later, at 192 min (3.2 hours). OD_max_ is followed by a turbidity decline (corresponding to culture-wide bacterial lysis [7]) that takes place until around 275 min (~4.5 hours). This adds up to a total duration of lysis inhibition in the range of 200 min, that is, over 3 hours after the point of Deviation. **Methods:** The bacterial host is *Escherichia coli* CR63. This culture was started from an overnight, 1 ml to 200 ml fresh broth, grown for one hour, and then cooled in an ice-water bath prior phage addition of an arbitrary titer of phages (though an amount insufficient to impede growth in culture turbidity over the first hour of incubation). The media used was trypticase soy broth as supplemented with 2.9 g/liter NaCl, the latter a known adsorption cofactor for phage T4 [33]. Data points were taken automatically every 4 min using a Molecular Devices Thermomax microtiter plate reader employing a 650 nm filter. The reader otherwise was set to 37°C, with shaking for 3 seconds prior to the taking of each data point. A Breathe-Easy sealing membrane (Excel Scientific) was placed over the 96-well plate to minimize contamination while still allowing reasonable oxygenation. See, e.g., Doermann [12] for similar-looking curves and also, e.g., Abedon [1,2,5]. An alternative perspective on this figure, better emphasizing the bacterial growth curve aspect (dotted line) can be found in Supplementary Materials A, Section A3.2. See Supplementary Materials B for the raw data used to generate this figure.

## A3.2 Lysis inhibition without and with emphasis on bacterial growth

Figure A3.1. Phage T4 lysis profile as generated in-house. The curves, dotted line for bacteria-only and solid for with phage, each represent only a single technical repeat (out of three, each done at the same time using the same microtiter plate and reader). This was done to better align the lower portions of their curves, allowing Deviation to be most apparent (Section 3.2, main text). The top curves differ from the bottom curves only in terms of the height of the *y* axis and the top panel otherwise is identical to Figure 1, main text (see there for methods). The two figures are presented here to illustrate the degree to which detail can be visually lost during illustrations of phage impact on the turbidity of bacterial cultures when emphasis is placed on bacterial population growth (lower panel) rather than on the phage impact on those bacteria (upper panel). This same point is made also with Figure A1.3, below.

# References

1. Abedon ST. Selection for lysis inhibition in bacteriophage. J Theor Biol*.* 1990;146:501-11.

2. Abedon ST. Lysis of lysis inhibited bacteriophage T4-infected cells. J Bacteriol*.* 1992;174:8073-80.

3. Abedon ST. Lysis and the interaction between free phages and infected cells. In: Karam JD, Kutter E, Carlson K, Guttman B, editors. *The Molecular Biology of Bacteriophage T4*.Washington, DC: ASM Press; 1994. 397-405.

4. Paddison P, Abedon ST, Dressman HK, Gailbreath K, Tracy J, Mosser E et al. The roles of the bacteriophage T4 r genes in lysis inhibition and fine-structure genetics: a new perspective. Genetics*.* 1998;148:1539-50.

5. Abedon ST. Bacteriophage T4 resistance to lysis-inhibition collapse. Genet Res*.* 1999;74:1-11.

6. Abedon ST. Bacteriophage intraspecific cooperation and defection. In: Adams HT, editor. *Contemporary Trends in Bacteriophage Research*.Hauppauge, New York: Nova Science Publishers; 2009. 191-215.

7. Abedon ST. Look who's talking: T-even phage lysis inhibition, the granddaddy of virus-virus intercellular communication research. Viruses*.* 2019;11:951.

8. Rajnovic D, Munoz-Berbel X, Mas J. Fast phage detection and quantification: An optical density-based approach. PLoS One*.* 2019;14:e0216292.

9. Kasman LM, Kasman A, Westwater C, Dolan J, Schmidt MG, Norris JS. Overcoming the phage replication threshold: a mathematical model with implications for phage therapy. J Virol*.* 2002;76:5557-64.

10. Hershey AD. Spontaneous mutations in bacterial viruses. Cold Spring Harbor Symposia on Quantitative Biology*.* 1946;11:67-77.

11. Underwood N, Doermann AH. A photoelectric nephelometer. Rev Scient Instr*.* 1947;18:665-72.

12. Doermann AH. Lysis and lysis inhibition with Escherichia coli bacteriophage. J Bacteriol*.* 1948;55:257-75.

13. Carlson K. Single-step growth. In: Karam JD, editor. *Molecular Biology of Bacteriophage T4*.Washington: ASM Press; 1994. 434-437.

14. Abedon ST. Further considerations on how to improve phage therapy experimentation, practice, and reporting: pharmacodynamics perspectives. Phage*.* 2022;3:98-111.

15. Poisson frequencies calculator. 2022 <http://www.phage.org/calculators/Poisson.html>

16. Freedman ML, Krisch RE. Enlargement of *Escherichia coli* after bacteriophage infection I. description of phenomenon. J Virol*.* 1971;8:87-94.

17. Freedman ML, Krisch RE. Enlargement of *Escherichia coli* after bacteriophage infection II. proposed mechanism. J Virol*.* 1971;8:95-102.

18. Delbruck M. Bacterial viruses or bacteriophages. Biol Rev*.* 1946;21:30-40.

19. Shymialevich D, Wojcicki M, Wardaszka A, Swider O, Sokolowska B, Blazejak S. Application of lytic bacteriophages and their enzymes to reduce saprophytic bacteria isolated from minimally processed plant-based food products-in vitro studies. Viruses*.* 2022;15:9.

20. Abedon ST, Hyman P, Thomas C. Experimental examination of bacteriophage latent-period evolution as a response to bacterial availability. Appl Environ Microbiol*.* 2003;69:7499-506.

21. Geng Y, Nguyen TVP, Homaee E, Golding I. Using bacterial population dynamics to count phages and their lysogens. Nat Commun*.* 2024;15:7814.

22. Krueger AP. A method for the quantitative determination of bacteriophage. J Gen Physiol*.* 1930;13:557-64.

23. Adams MH. *Bacteriophages*. New York: InterScience; 1959.

24. Maillard JY, Beggs TS, Day MJ, Hudson RA, Russell AD. The use of an automated assay to assess phage survival after a biocidal treatment. J Appl Bacteriol*.* 1996;80:605-10.

25. Maillard JY, Russell AD. Viricidal activity and mechanisms of action of biocides. Sci Prog*.* 1997;80:287-315.

26. Turner PE, Draghi JA, Wilpiszeski R. High-throughput analysis of growth differences among phage strains. J Microbiol Meth*.* 2012;88:117-21.

27. Müller J. *Quantification of microbial fitness: costs of protein overexpression and phage infection*. Universität zu Köln; 2025.

28. Blazanin M, Vasen E, Vilaró Jolis C, An W, Turner PE. Theoretical validation of growth curves for quantifying phage-bacteria interactions. bioRxiv 2023 https://doi.org/10.1101/2023.06.29.546975

29. Blazanin M, Vasen E, Vilaró Jolis C, An W, Turner PE. Quantifying phage infectivity from characteristics of bacterial population dynamics. Proc Natl Acad Sci U S A*.* 2025;122:e2513377122.

30. Dalmasso M, de HE, Neve H, Strain R, Cousin FJ, Stockdale SR et al. Isolation of a novel phage with activity against *Streptococcus mutans* biofilms. PLoS One*.* 2015;10:e0138651.

31. Xie Y, Wahab L, Gill JJ. Development and validation of a microtiter plate-based assay for determination of bacteriophage host range and virulence. Viruses*.* 2018;10:189.

32. Storms ZJ, Teel MR, Mercurio K, Sauvageau D. The virulence index: a metric for quantitative analysis of phage virulence. Phage (New Rochelle )*.* 2020;1:27-36.

33. Puck TT, Garen A, Cline J. The mechanism of virus attachment to host cells. I. The role of ions in the primary reaction. J Exp Med*.* 1951;93:65-88.
