# Supplementary figures and images for: Bacteriophage titering by optical density means: KOTE assays
Source: Open Life Sci. 2025 Dec 30;20(1):20251209. doi: 10.1515/biol-2025-1209 (PMC13011615; doi:10.1515/biol-2025-1209)

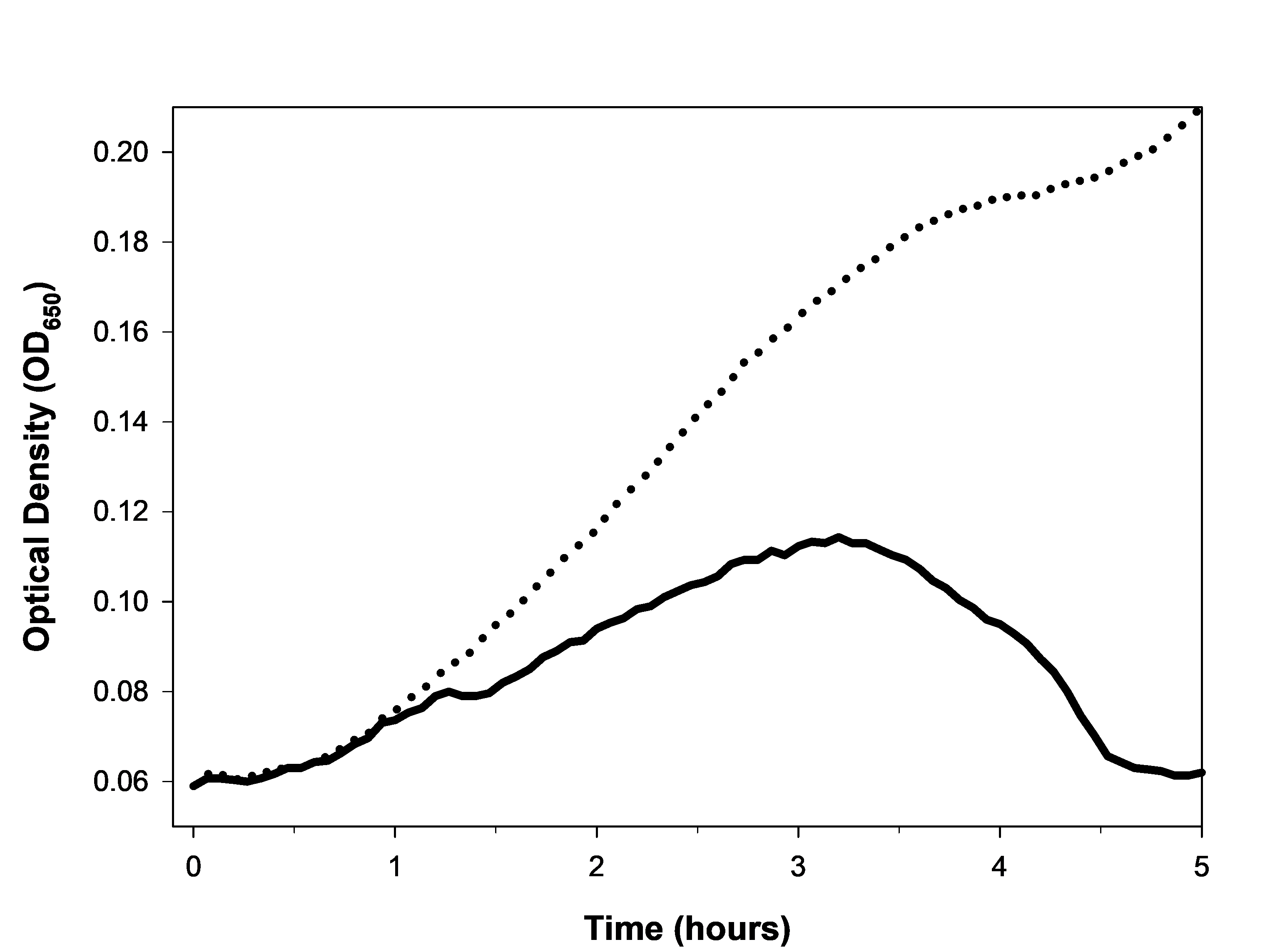

Supplement: Supplementary file 7 — Supplementary Material [file j_biol-2025-1209_suppl_007.zip › j_biol-2025-1209_suppl_007.TIF]

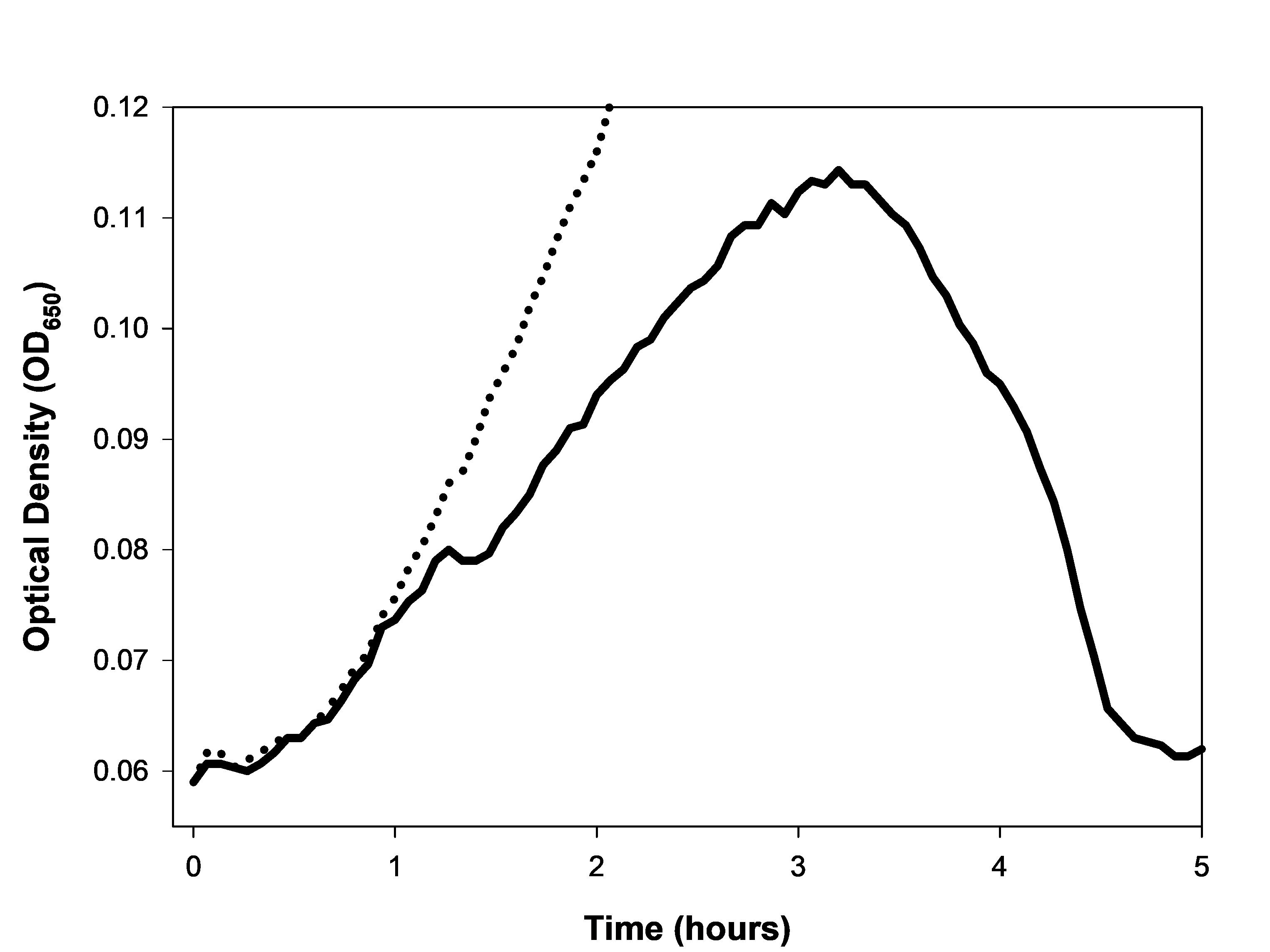

Supplement: Supplementary file 8 — Supplementary Material [file j_biol-2025-1209_suppl_008.zip › j_biol-2025-1209_suppl_008.TIF]
