# Supplementary material for: Bacteriophage titering by optical density means: KOTE assays
Source: Open Life Sci. 2025 Dec 30;20(1):20251209. doi: 10.1515/biol-2025-1209 (PMC13011615; doi:10.1515/biol-2025-1209)
Supplement: Supplementary file 9 — Supplementary Material [file j_biol-2025-1209_suppl_009.docx]

# Supplementary materials

Supplementary Materials A: Narrative summary of Rajnovic *et al.* second figure (A1), narrative summary of some history of KOTE-like assays (A2), and alternative perspective on the Figure 2 lysis inhibition curve (A3).

Supplementary Materials B: Data used to generate Figure 2.

Supplementary Materials C: Rajnovic *et al.* optical density data with calculations.

Supplementary Materials D: Geng *et al.* optical density data with calculations.

Supplementary Materials E: Geng *et al.* supplementary optical density data with calculations.

Supplementary Materials F: Summary from Supplementary Materials C, D, and E; basis for Table 2.
